# Untargeted LC/HRMS Metabolomics Analysis and Anticancer Activity Assay on MCF-7 and A549 Cells from *Coleus amboinicus* Lour Leaf Extract

**DOI:** 10.5812/ijpr-143494

**Published:** 2024-04-20

**Authors:** Kasta Gurning, Suratno Suratno, Endang Astuti, Winarto Haryadi

**Affiliations:** 1 Department of Chemistry, Faculty of Mathematics and Natural Sciences, Universitas Gadjah Mada, 55281, Yogyakarta, Indonesia; 2 Department of Pharmacy, Sekolah Tinggi Ilmu Kesehatan Senior Medan, 20141, Medan, Indonesia; 3 Research Center for Food Technology and Processing (PRTPP), National Research and Innovation Agency (BRIN), 55861, Yogyakarta, Indonesia

**Keywords:** *Coleus amboinicus*, Metabolomics, A549 Cells, MCF-7 Cells, LC/HRMS

## Abstract

**Background:**

Cancer remains the leading cause of death globally, with breast cancer being the foremost cause among women and lung cancer ranking second for both women and men.

**Objectives:**

This study aimed to identify the metabolomic content of *Coleus amboinicus* leaves and evaluate their anticancer activities against breast and lung cancer cells, thereby providing insights into potential alternative treatments for these cancers and initiating research on active isolates from *C. amboinicus* leaves.

**Methods:**

The research methodology involved maceration using ethanol, followed by multistage partitioning with solvents n-hexane, chloroform, and ethyl acetate. Phytochemical screening was performed using standard reagents to detect the presence of alkaloids, phenolics, polyphenols, flavonoids, steroids/triterpenoids, and saponins. Metabolomic profiling was conducted using LC/HRMS, and the anticancer activities against lung cancer cells (A549) and breast cancer cells (MCF-7) were assessed using the MTT assay.

**Results:**

The results showed that the *C. amboinicus* extract contains various secondary metabolite groups such as alkaloids, phenolics and polyphenols, flavonoids, steroids, triterpenoids, and saponins.

**Conclusions:**

The diverse metabolomic profile of the *C. amboinicus* leaf extract demonstrated potential activity against cancer, as evidenced by in vitro tests on lung (A549) and breast (MCF-7) cancer cells. *C. amboinicus* leaf extract shows promise as an active ingredient in the prevention and alternative natural treatment of lung and breast cancer. Further research and testing, both in vivo and clinically, are warranted.

## 1. Background

The *Coleus amboinicus* plant, known as Lour, holds significant potential as a source of food and medicinal ingredients. Among the Batak tribe, it is referred to as bangun-bangun leaves and is commonly consumed as a vegetable. Beyond its nutritional value, *C. amboinicus* is rich in secondary metabolite compounds, making it a promising candidate for pharmaceutical applications. These compounds include phenolics, polyphenols, flavonoids, diterpenes, coumarins, diterpenoids, and alkaloids (1, 2), which have demonstrated potential as antioxidants (3, 4), anticancer agents, antiproliferatives, and cytotoxics (5).

Cancer ranks among the top three causes of death worldwide for both men and women, with breast cancer being the leading cause of death among women, and lung cancer being the second leading cause for both genders. Conventional treatments for these cancers typically involve chemotherapy, surgery, radiation, targeted therapy, and immunotherapy (6, 7). However, current chemotherapy regimens, which often employ combinations of drugs acting through different mechanistic pathways on tumor cells, have shown limited efficacy and are associated with significant side effects, including drug resistance and toxicity. This can lead to a decrease in quality of life and survival rates (8). Similarly, radiation therapy, although used in conjunction with chemotherapy, can have severe side effects. The use of radiation in thoracic therapy can lead to a range of complications, such as restrictive cardiomyopathy, coronary artery disease, pericardial disease, and heart failure with either preserved or reduced ejection fraction (9, 10).

The current treatment methods for lung and breast cancer are often found to be ineffective. As a result, there is a significant interest in exploring the potential of active metabolic compound derivatives (bioactive compounds) extracted from *C. amboinicus* leaves for treating these cancers. LC/HRMS offers a promising approach for the discovery of detailed metabolites and metabolomics by detecting both primary and minor constituents in natural extracts. This technology provides high sensitivity and efficiency, enabling the identification and confirmation of the chemical structures of metabolite compounds in plants. The efficacy of this plant species in various treatments has been documented (11).

Utilizing active compounds from plants is anticipated to benefit 50 – 60% of cancer patients, offering an alternative therapy for the treatment of various cancers and other diseases (12). The adoption of natural agents and their derivatives is seen as a superior method for disease prevention and treatment (13).

## 2. Objectives

Given this context, the current study aims to evaluate the potential activity of *C. amboinicus* leaf extract against breast cancer cells (MCF-7) and lung cancer cells (A549) using the MTT assay and to identify potential metabolite content with LC/HRMS instruments.

## 3. Methods

### 3.1. Materials

The study utilized *C. amboinicus* leaves, 3-(4, 5-dimethylthiazol-2-yl)-2, 5-diphenyltetrazolium bromide (MTT), Dimethyl sulfoxide (DMSO) from Merck (D1435), Trypsin-EDTA, Elisa microplate readers, ethanol, n-hexane, chloroform, ethyl acetate (all pro analysis grade), cisplatin (EDQM C22100000), doxorubicin, antibiotics (Sigma), 96-well plates (Merk Nest 701001), Phosphate Buffered Saline (PBS) (Gibco 18912-014), PrestoBlue™ Cell Viability Reagent (Thermofisher A132), Roswell Park Memorial Institute (RPMI-1640) and DMEM-H medium (Gibco 11875-093), Fetal Bovine Serum (FBS) (Gibco 10270-106).

### 3.2. Preparation and Extraction of C. amboinicus Leaves

*Coleus amboinicus* was sourced from Sosor Ladang Village, Toba Regency, North Sumatra Province, Indonesia, and authenticated by a botanist (voucher number 0220/S.Tb./I/2023). Fresh green leaves were cleaned under running water, air-dried, and subsequently dried in an oven at 50°C. The dried leaves were ground into a powder. Ethanol was used as the solvent for the maceration extraction over three days. The maceration filtrate was strained using Whatman No. 1 filter paper. The marc was re-macerated under identical conditions. The ethanol extract of *C. amboinicus* was concentrated under reduced pressure using a rotary evaporator at 55°C. Part of this concentrated ethanol extract underwent further partitioning with solvents of varying polarities in a separating funnel, starting with n-hexane, followed by chloroform, and finally ethyl acetate. This process yielded initial ethanol extract, n-hexane extract, chloroform extract, ethyl acetate extract, and the remaining ethanol extract, which were utilized in subsequent stages (3, 4).

### 3.3. Phytochemical Screening

Phytochemical screening was conducted on the five extracts utilizing standard reagents. This screening aimed to qualitatively identify the presence of bioactive compounds in each extract, including phenolics and polyphenols, steroids, triterpenoids, saponins, and alkaloids (14, 15).

### 3.4. Metabolomics Preparation and Analysis

Fifty milligrams of each dry extract (initial ethanol, n-hexane, chloroform, ethyl acetate, and residual ethanol) were placed into 2 mL centrifuge tubes. One milliliter of LC-MS-grade methanol was added to each tube, followed by vortexing for 1 minute. The samples were then centrifuged at 5000 rpm for 10 minutes. The analysis was performed using liquid chromatography and high-resolution orbitrap spectrometry, adopting methods from prior research. The chromatography column measured 100 mm x 2.1 mm ID x 2.6 µm. The mobile phase consisted of water with 0.1% formic acid (A) and ethanol with 0.1% formic acid (B), both of MS-grade, at a flow rate of 0.3 mL/minute. Untargeted metabolomic compound screening for each extract was performed using full MS/dd-MS^2 acquisition mode in either positive or negative polarity/ionization states (16, 17).

### 3.5. Preparation and Testing of Anticancer Activity in Vitro

#### 3.5.1. Cell Culture

Breast cancer cells (MCF-7) and lung cancer cells (A549) were maintained at the Integrated Laboratory of Padjadjaran University, Bandung, Indonesia. The RPMI and DMEM-H culture media were prepared, containing 10% PBS and 50 µm/50 mL antibiotics. The cell cultures were incubated at 37°C and 5% CO_2_ until they reached a minimum growth percentage of 70%.

#### 3.5.2. Cytotoxicity Tests Against Cancer Cells

The cultured MCF-7 and A549 cells were advanced to the cytotoxicity testing stage, utilizing various concentrations of each extract (7.81; 15.63; 31.25; 62.5; 125; 250; 500; and 1000 µg/mL) dissolved in DMSO. Cisplatin was employed as the positive control for breast cancer cell testing, and doxorubicin for lung cancer cells. The methodology was based on procedures outlined in prior research (18, 19).

## 4. Results and Discussion

### 4.1. Phytochemical Screening

A total of 1.14 kg of *C. amboinicus* leaf powder was extracted using ethanol, yielding 108.01 ± 0.01 g of concentrated ethanol extract. Phytochemical screening with standard reagents provided qualitative insights into the bioactive compounds present in each extract (Table 1). 

**Table 1.** Results of Phytochemical Screening of *C. amboinicus* Extract

No	Phytochemical Components	Reagent	Initial Ethanol Extract	Extract Partition			
				n-Hexane	Chloroform	Ethyl Acetate	Residual Ethanol
**1**	Flavonoids	Shinoda test	+	-	-	+	+
**2**	Phenolics and polyphenolics	FeCl_3_ 5% in ethanol	+	-	+	+	+
**3**	Alkaloids	Dragendroff	+	-	+	-	+
		Mayer	+	-	+	-	+
		Wagner	+	-	-	-	+
**4**	Triterpenoids/steroids	Liebermann-Buchard	+	+	+	+	+
**5**	Saponins	Foaming test	+	+	+	-	+

Abbreviations: +, present; -, non-present.

The presence of diverse bioactive compounds in *C. amboinicus* leaf extract, including alkaloids, diterpenes, triterpenes, and polyphenols, suggests potential pharmacological activities (1, 20). These secondary metabolite compounds, such as alkaloids, diterpenes, triterpenes, and polyphenols have shown anticancer potential in various studies and are utilized therapeutically (21). The diverse secondary metabolites in *C. amboinicus* leaf extract correlate positively with its potential anticancer properties.

### 4.2. Metabolomic Analysis of C. amboinicus Extract Using LC/HRMS

**Figure 1.**
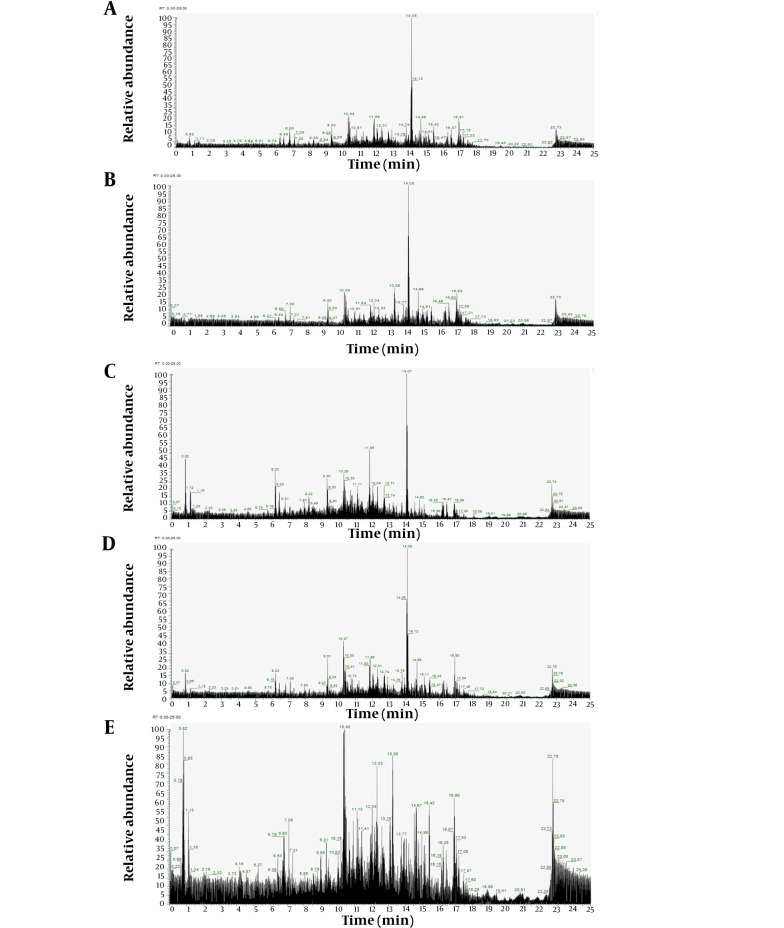
Chromatogram analysis of *C. amboinicus* leaf extract. A, initial ethanol; B, n-hexane; C, chloroform; D, ethyl acetate; E, residual ethanol.

The LC/HRMS analysis employed the Ultimate 3000 RSLC system coupled with a Fusion Lumos Tribrid orbital mass spectrometer. Compounds were ionized using a heated electrospray ionization source in positive mode. A comprehensive MS spectrum was acquired on the Orbitrap at a resolution of 120,000, spanning a mass range of m/z 160 - 2000. The four most intense ions from the full scan, within the mass range of m/z 400 - 2000, underwent fragmentation both in collision-induced dissociation (CID) mode and higher-energy C-trap dissociation (HCD) mode. This approach enhances the capability to analyze complex sample matrices, achieving separations 5 - 10 times more rapidly than conventional methods (22, 23). Utilizing the LC/HRMS instrument represents an advanced method with heightened sensitivity and efficiency. Furthermore, it facilitates the identification of a detailed metabolomic profile, including both primary and minor constituents in natural extracts. The metabolomic profile of each *C. amboinicus* extract via LC/HRMS is novel information, crucial for advancing research. The chromatogram analysis results for each *C. amboinicus* leaf extract using LC/HRMS are presented in Figure 1. 

The LC/HRMS analysis results for the initial ethanol extract identified 10 compounds based on their peak areas, as detailed in Table 2. The top three constituents of the initial ethanol extract, from a total of 284 identified components, were: (1) 5,6-dihydroxy-2,6-bis(3-methyl-2-buten-1-yl)-4-(4-methylpentanoyl)-4-cyclohexene-1,3-dione (C_22_H_32_O_5_; 35.706%), (2) gibberellin A24 (C_20_H_26_O_5_; 10.024%), and (3) NP-020713 (C_20_H_26_O_4_; 4.096%). The LC/HRMS analysis of the extracts obtained from partitioning the initial ethanol extract revealed the top 10 compounds by peak area for each extract (Table 3). For the n-hexane extract, from 210 identified components, the top three were: (1) (4S,5E)-4-Hydroxy-6-{(1S,2R,5S)-5-hydroxy-3-oxo-2-[(2Z,5Z,8Z)-2,5,8-undecatrien-1-yl]cyclopentyl}-5-hexenoic acid (C_22_H_32_O_5_; 48.466%), (2) 1-(4-Hydroxy-3-methoxyphenyl)-3,5-decanediyl diacetate (C_21_H_32_O_6_; 7.177%), and (3) 5-Pentylresorcinol (C_11_H_16_O_2_; 3.464%). For the chloroform extract, out of 325 identified components, the leading three were: (1) resolvin D1 (C_22_H_32_O_5_; 13.485%), (2) 5-Pentylresorcinol (C_11_H_16_O_2_; 6.887%), and (3) NP-005870 (C_20_H_26_O_5_; 5.892%). The ethyl acetate extract, with 264 identified components, was dominated by: (1) Resolvin D1 (C_22_H_32_O_5_; 40.878%), (2) gibberellin A24 (C_20_H_26_O_5_; 7.239%), and (3) NP-020713 (C_20_H_26_O_4_; 4.927%). For the residual ethanol extract, from 293 identified components, the top three were: (1) choline (C_5_H_13_NO; 6.639%), (2) 9-HOTE (C_18_H_30_O_3_; 5.224%), and (3) (1Z)-1-(4-Hydroxy-3-methoxyphenyl)-1-dodecene-3,5-dione (C_19_H_26_O_4_; 3.653%).

**Table 2.** Metabolomics Components of the Initial Ethanol Extract with the Highest Peak Area

No	Initial Ethanol Extract			
	Compounds	Formula	Group Area	Ionization (ESI)
**1**	5,6-Dihydroxy-2,6-bis(3-methyl-2-buten-1-yl)-4-(4-methylpentanoyl)-4-cyclohexene-1,3-dione	C_22_H_32_O_5_	19497374364	-
**2**	gibberellin A24	C_20_H_26_O_5_	5473876018	-
**3**	NP-020713	C_20_H_26_O_4_	2236370277	+
**4**	NP-005870	C_20_H_26_O_5_	2005457620	-
**5**	5-Pentylresorcinol	C_11_H_16_O_2_	1835585634	+
**6**	Arachidonic acid	C_20_H_32_O_2_	1764346397	+
**7**	13(S)-HOTrE	C_18_H_30_O_3_	1452905769	-
**8**	haplophytine	C37H_40_N_4_O_7_	1024190439	+
**9**	2-Hydroxy-3-(phosphonooxy)propyl (9Z,12Z,15Z)-9,12,15-octadecatrienoate	C_21_H_37_O_7_P	745059283	-
**10**	9S,13R-12-Oxophytodienoic acid	C_18_H_28_O_3_	603455078	+

**Table 3.** Metabolomics of Each Extract Resulting from Partitioning from Ethanol Extract Based on the Highest Peak Area

No	n-Hexane Extrac				Chloroform Extract			
	Compounds	Formula	Group Area	Ionization (ESI)	Compounds	Formula	Group Area	Ionization (ESI)
**1.**	(4S,5E)-4-Hydroxy-6-{(1S,2R,5S)-5-hydroxy-3-oxo-2-[(2Z,5Z,8Z)-2,5,8-undecatrien-1-yl]cyclopentyl}-5-hexenoic acid	C_22_H_32_O_5_	20872483347	+	Resolvin D1	C_22_H_32_O_5_	6324859959	+
**2.**	1-(4-Hydroxy-3-methoxyphenyl)-3,5-decanediyl diacetate	C_21_H_32_O_6_	3090632646	-	5-Pentylresorcinol	C_11_H_16_O_2_	3230461664	+
**3**	5-Pentylresorcinol	C_11_H_16_O_2_	1491643393	+	NP-005870	C_20_H_26_O_5_	2763718431	-
**4**	haplophytine	C_37_H_40_N4O_7_	1471009433	+	NP-020713	C_20_H_26_O_4_	2436462762	+
**5**	Arachidonic acid	C_20_H_32_O_2_	960872349	+	13(S)-HOTrE	C_18_H_30_O_3_	1659921023	-
**6**	Pheophorbide A	C_35_H_36_N_4_O_5_	900790852	+	Stearidonic acid	C_18_H_28_O_2_	1524021068	+
**7**	9-HOTE	C_18_H_30_O_3_	810001605	-	4-Indolecarbaldehyde	C_9_H_7_NO	1353428049	+
**8**	Stearidonic acid	C_18_H_28_O_2_	627201677	+	NP-017061	C_20_H_30_O_4_	1330723133	+
**9**	Linolenic acid ethyl ester	C_20_H_34_O_2_	612428711	+	NP-005870	C_20_H_26_O_5_	1006625676	-
**10**	α-Linolenic acid	C_18_H_30_O_2_	556204914	+	Paracetamol	C_8_H_9_NO_2_	774182422	+
	**Ethyl acetate extract**				**Residual ethanol extract**			
**1.**	Resolvin D1	C_22_H_32_O_5_	19855216920	+	Choline	C_5_H_13_NO	2066589120	+
**2.**	gibberellin A24	C_20_H_26_O_5_	3516227224	-	9-HOTE	C_18_H_30_O_3_	1626215750	-
**3**	NP-020713	C_20_H_26_O_4_	2393343023	+	(1Z)-1-(4-Hydroxy-3-methoxyphenyl)-1-dodecene-3,5-dione	C_19_H_26_O_4_	1137046653	-
**4**	Hexyl 2-furoate	C_11_H_16_O_3_	1927943357	+	3,3'-Diisopropyl-6,6'-dimethyl-2,2',5,5'-biphenyltetrol	C_20_H_26_O_4_	1065773928	-
**5**	13(S)-HOTrE	C_18_H_30_O_3_	1803807869	-	1,2,3,4-Tetramethyl-1,3-cyclopentadiene	C_9_H_14_	972978432	+
**6**	NP-005870	C_20_H_26_O_5_	1604081151	-	6-Hydroxy-3,4a,5-trimethyl-2-oxo-2,4,4a,5,6,7,8,8a,9,9a-decahydronaphtho[2,3-b]furan-4-yl (2Z)-2-methyl-2-butenoate	C_20_H_28_O_5_	923282617	-
**7**	Arachidonic acid	C_20_H_32_O_2_	948801732	+	2-Hydroxy-3-(phosphonooxy)propyl (9Z,12Z,15Z)-9,12,15-octadecatrienoate	C_21_H_37_O_7_P	810505519	-
**8**	4-Indolecarbaldehyde	C_9_H_7_NO	553570860	+	Caffeic acid	C_9_H_8_O_4_	708108701	-
**9**	8-{3-Oxo-2-[(2E)-2-penten-1-yl]-1-cyclopenten-1-yl}octanoic acid	C_18_H_28_O_3_	540453342	+	3,4-Methylenesebacic acid	C_12_H_18_O_4_	677578291	-
**10**	Asiatic acid	C_30_H_48_O_5_	505413139	-	Arachidonic acid	C_20_H_32_O_2_	573104530	+

### 4.3. Cytotoxicity of C. amboinicus Leaf Extract

The anticancer activity of each extract was assessed using the MTT assay, a colorimetric technique evaluating mitochondrial cell dehydrogenase enzyme activity. In this assay, the conversion of tetrazole (yellow) to formazan (purple) occurs in living cells, indicating metabolic activity. If the cells are deceased, the enzymatic reaction does not proceed, and the solution remains yellow (24). The anticancer efficacy of each extract was tested in vitro on lung cancer cells (A549) and breast cancer cells (MCF-7). The efficacy of each extract against these cancer cell types is summarized in Table 4. 

**Table 4.** Testing the Anticancer Activity of *C. amboinicus* Leave Extract Using the MTT Method

No	Treatment	IC_50_, µg/mL		
		Lung Cancer Cells (A549)	Breast Cancer Cells (MCF-7)	Normal Cells (CV-1)
**1.**	Initial ethanol extract	212.80	154.80	501.19
**2.**	n-hexane extract	544.80	217.20	630.96
**3.**	Chloroform extract	251.20	102.30	189.10
**4.**	Ethyl acetate extract	554.60	108.00	457.09
**5.**	Residual ethanol extract	554.10	396.40	531.00
**6.**	Cisplatin	-	53 ^a^	-
**7.**	Doxorubicin	2.1 ^a^	-	-

^a^ The anticancer activity value is expressed in µM units.

**Figure 2.**
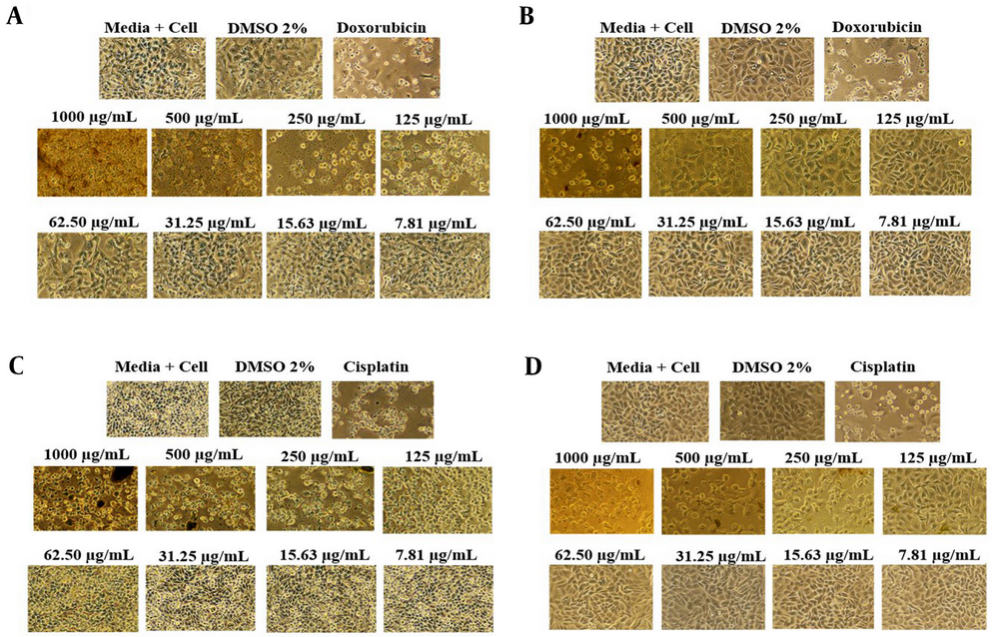
Anticancer activity testing results for the most effective extracts: A, initial ethanol extract against lung cancer cells (A549); B, initial ethanol extract on normal cells (CV-1); C, chloroform extract against breast cancer cells (MCF-7); D, chloroform extract on normal cells (CV-1).

**Figure 3.**
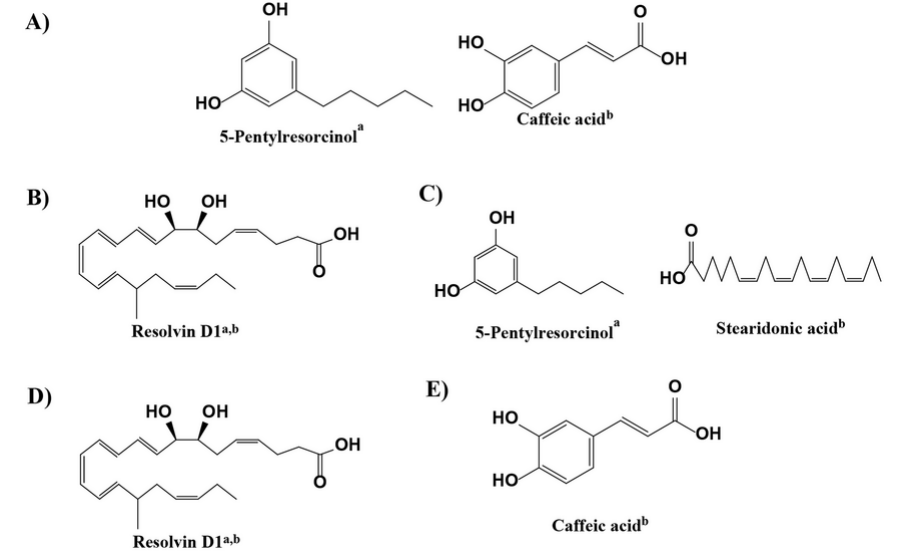
Compounds with the highest peak area from each *C. amboinicus* leaf extract that correlate with potential anticancer activity against breast and lung cancer: A, initial ethanol; B, n-hexane; C, chloroform; D, ethyl acetate; and E, residual ethanol.

According to Table 4, the initial ethanol extract exhibits more potent anticancer activity (IC_50_) against MCF-7 cells (154.80 µg/mL) compared to A549 cells (212.80 µg/mL) and displays lower cytotoxicity towards normal cells (501.19 µg/mL) (Figure 2). The examination of extracts derived from partitioning the initial ethanol extract revealed that the anticancer activity in MCF-7 cells was more pronounced than in A549 cells, with lower cytotoxic effects on normal cells, except for the chloroform extract's cytotoxic properties. As per the National Cancer Institute (NCI) classification of cytotoxic activity values for the initial ethanol extract, the chloroform extract falls into the weak category, whereas the n-hexane, ethyl acetate, and residual ethanol extracts are considered inactive against lung cancer cells (A549). In contrast, all extracts exhibit weak anticancer activity against breast cancer cells (MCF-7) and are non-cytotoxic to normal cells, except for the chloroform and ethyl acetate extracts. The NCI categorizes cytotoxic activity based on the IC_50_ against cancer cells as follows: IC_50_ < 20 μg/mL is classified as high, IC_50_ 20 - 100 μg/mL as medium, IC_50_ 101 - 500 μg/mL as weak, and IC_50_ > 500 μg/mL as inactive regarding anticancer potential (25). Metabolomic compounds with the largest peak area are directly associated with the anticancer activity against breast (MCF-7) and lung cancer (A549) for each extract (Figure 3). 

### 4.4. Conclusions

The *C. amboinicus* leaf extract is rich in various metabolites, including phenolic and polyphenol groups, flavonoids, alkaloids, steroids, triterpenoids, and saponins. Furthermore, this plant extract demonstrates potential anticancer activity against lung (A549) and breast (MCF-7) cancer cells. Notably, the initial ethanol extract showed the most potent activity against lung cancer cells (A549) with an IC_50_ value of 212.80 µg/mL, while the chloroform extract, obtained through sequential partitioning, was most effective against breast cancer cells (MCF-7) with an IC_50_ value of 102.30 µg/mL. Further isolation and testing are essential to identify active compounds effective against lung (A549) and breast (MCF-7) cancer cells.

## Data Availability

The dataset presented in the study is available on request from the corresponding author during submission or after publication.
